# The American and Colonial Journals: Medicine

**Published:** 1839-01

**Authors:** 


### MEDICINE.

### *Observations on Cerebral Auscultation.* By J. D. FISHER, M.D.

[IN the year 1833, Dr. Fisher read a paper before the Boston Medical Society, on what he called the *cephalic bellows-sound,* which he has discovered to accompany certain diseases of the brain. The present paper, also read before the Boston Society, is an exposition of his more extended views and experience on the same subject, the more important of which we shall here extract. We know nothing personally of this new sign; and we lay the statements of Dr. F. before our readers without a comment.]

*Normal sounds.* I can, by applying my ear to the heads of healthy children, hear a sound which is evidently produced by the impinging of the air against the walls of the nasal cavities during the act of respiration. It commences and terminates with the respiratory act. This sound is peculiar, and is readily recognized. It is the one which first attracts the attention, and resembles in all respects, except in intensity, the respiratory murmur caused by the air passing through the nostrils when the mouth is closed, and which is then audible to the person breathing. This sound, which I would denominate *the cephalic sound of respiration*, is heard rather more distinctly during expiration than inspiration; and becomes somewhat modified when the membrane of the nose is affected by a cold or other cause. A second sound which strikes the ear is one whose impulse seems to be transmitted from a distance. It is evidently that of the heart, and is a soft mellow sound, resembling that produced by softly palpipating our cheeks when distended by air. It corresponds with the action of the heart, and varies in frequency and intensity as the contraction of that organ varies in rapidity and power. It may be called *the cephalic sound of the heart*. The cephalic sound of respiration and the cephalic sound of the heart are the only sounds which auscultation discovers in the heads of healthy children when they are asleep or at perfect rest. If, however, the child should cry, or speak, or swallow, whilst the ear is applied upon its head, then other sounds may be heard. When the child cries or speaks, the sound of its voice is very distinctly heard at the surface of its head, or on whatever part of it the ear may be placed. It is generally sharp and piercing, and seems to arise out of the cranium itself, so near does it appear to be to the ear; and, when it is heard through the stethoscope, it seems as if it were vibrating about the mouth, and even to pass into the canal, of the instrument. This sound I would term *the cephalic sound of the voice*. It varies somewhat in its tone and apparent approximation to the ear, at different parts of the head. At the unclosed fontanelle it is less sharp, and somewhat more mellow and diffusive in its character, than at any other part of the head, and seems to be further removed from the surface. The other sound which attracts the attention attends the act of deglutition. When a child swallows any fluid, a sound of a compound character is readily distinguished by applying the ear to its head. I hardly know what the sound resembles, or to what I can compare it. It is peculiar, and cannot be described; but it can never be mistaken for any other bruit after it has once been observed and recognized. It has a liquid and a dull, massive tone, and is evidently caused by the act of deglutition: I shall therefore denominate it *the cephalic sound of deglutition*. This last-named sound may be best noticed while a child is nursing; for then it is less liable to be obscured or masked by the cephalic sounds of respiration or by any movements of the head.

I have described these sounds as they are developed in the heads of infants previous to the closure of the anterior fontanelle. They become modified in some respects by the influence of growth, and the density of the brain and cranium: this is more strikingly the case with the cephalic sound of the heart. In early infancy, and before the period of dentition, the cephalic sound of the heart is distinguished by a softness and diffusiveness of tone which it does not possess afterwards. In youths and adults, the sound acquires a coarser and harsher tone, and seems to be more remote from the ear. The cephalic sounds of the voice and deglutition are not so sensibly affected by the growth and increased density of the cranium and its contents. All the sounds which I have now described are most distinctly heard at the summit of the cranium, although they may be easily detected at any portion of its surface. Such are the murmurs or bruits which are constantly occurring in or traversing the heads of healthy individuals, and which auscultation reveals and enables us to appreciate. They unquestionably are the results of the functions to which I have referred them.

These cerebral murmurs, I find from observation, become modified by the presence of certain diseases within the cranium, and thus become symptoms of cerebral affections. This is manifestly the case with the cephalic sound of the heart. The cephalic bellows-sound which, in 1832, I discovered to accompany certain diseases of the brain is a modification of the cephalic sound of the heart.

*Morbid sounds.* The most important of these is that first described by Dr. Fisher, and still named by him *the cephalic bellows-sound.* It is thus described in his first case:

On applying my ear over the anterior fontanelle, which I was induced to do from observing its strong pulsatory motions, I heard a distinct bellows-sound. The sound is coarse, abrupt, rasp-like; is synchronous with the pulsatory motions of the fontanelle and with the arterial pulse, and occurs 144 times in a minute. It can be heard over any portion of the cranium, but it is most distinct at the anterior fontanelle. While listening to this sound, I can hear a murmur accompanying his respiration, and also a sharp resonance of his voice when he cries or utters any vocal tones. These sounds are distinct from and independent of the bellows-sound and of each other, and are constant in their occurrence. This new auscultic symptom, which may be properly called the cephalic bellows-sound, is confined to the head, as nothing resembling it can be detected in the heart or great blood-vessels passing from it, or in any artery or organ below or exterior to the head. . . .

This new auscultic symptom is not a phenomenon of health. It cannot be detected in the heads of children or adults who are free from all disease or derangement of the bodily functions; while, on the other hand, it has been found to accompany, 1st, chronic hydrocephalus; 2d, congestion of the cerebral organs; 3d, acute inflammation of the cerebral organs, with serous effusion in or around them; 4th, abscesses in the brain; 5th, induration of the brain with effusion into its ventricles and at its base; 6th, compression of the brain. We have, then, one auscultic sound in the head which is a symptom of cerebral disease; and it is possible that the cephalic sounds of the voice and deglutition, as well as that of the heart, may also have been modified or altered in the same cases. I regret I did not devote more attention to these sounds, in order to ascertain the fact. Whether any alteration in the last-named normal cerebral sounds takes place or not, that which I have described under the name of cephalic bellows-sound accompanied, and was unquestionably dependent on, a pathological condition of the organs within the cranium. The whole history of the cases proves this; and we come now to a consideration of its seat and immediate cause; or, in other words, to enquire, 1st, in what organ or organs did this sound originate in the instances above mentioned? 2dly, what part of the cranium did it proceed from? and, 3dly, what was the immediate or proximate cause of its production? In regard to the organ or organs in which the sound had its origin, it is very evident, I think, that it originated and was seated in the arteries: for, in the first place, the sound was distinct from that produced by respiration, by deglutition, or any other operation going on within the head, that we can conceive of, save arterial action. Secondly, it was synchronous with the pulsations and impulse of the heart and of the carotid and temporal arteries, and also with the rising and impulse of the brain, as observed by placing the finger upon the unclosed fontanelle. Thirdly, the sound ceased, or at any rate was rendered inaudible, by compressing the carotid arteries, and arresting the circulation of the blood through them; and it became fainter and less distinct as the patient grew weak and the arterial action feeble. Fourthly, it resembled in all respects the *bruit de soufflet* which we hear in diseases of the heart and of the arteries; and, like that, it often passed into a continuous murmur, and was characterized at times by a musical tone. Fifthly, in studying the structure, distribution, and functions of the organs inclosed by the cranium, we must, I think, be convinced that the arteries were the only organs which could have emitted a bellows-sound like that I have noticed. Assuming it as proved, then, that the sound in question proceeded from the arteries, I may further observe, that those situated at the base of the brain were probably the ones in which it originated. .

If, then, the bellows-sound proceeded from the arteries at the base of the brain, its production in the cases above related may be rationally and satisfactorily accounted for. It is now a well-established fact that the bellows-sound of the heart and of the arteries arises from an impediment to the flow of the blood through these organs. An impediment to the free passage of the blood through the large arteries which lay on the base of the skull must, I conceive, have existed in these cases: for the brain is contained in a strong and unyielding bony case, and in itself incompressible. In all the cases in which the cephalic bellows-sound was heard, at least in all those in which it was heard and of which an autopsic examination was made, there was fluid congestion of blood-vessels, or a pathological state of the organs within the cranium which would and must have displaced the brain, and forced it against the compressible arteries on which it rested. The arteries being thus forced and pressed against the bony channels through which they coursed, their caliber must have been diminished. This condition of the arteries formed an impediment to the free passage of blood through them, and constituted the immediate or proximate cause of the cephalic bellows-sound. . .

In the course of the last three years I have noticed a modification of the normal cephalic sound of the heart in six cases of cerebral apoplexy. In each of these cases the sound of the heart, as heard at the surface of the cranium, was decidedly abnormal. Instead of its being soft, and appearing as if it proceeded from a distance, as in healthy adults, it seemed to be near the ear, and was characterized by a kind of impulse, as if the whole brain was suddenly raised up against the calvarium. So characteristic did this sound appear, I could not but believe that the brain *en masse* did actually strike against the cranium beneath my ear. . .

The sound, I am well aware, will not be easily detected and recognized by one who has had no experience in cerebral auscultation; but, having made himself familiar with the normal cephalic sounds, and particularly with the cephalic sound of the heart, the auscultator will meet with little or no difficulty in distinguishing the impulsive sound under consideration when he auscultates the heads of those labouring under cerebral apoplexy. I have heard it in every case of the affection in which I have practised cerebral auscultation; and from this fact I am strongly inclined to believe that it is a constant symptom of the disease.

Indeed, when we consider the condition of the brain and of the arteries at its base, resulting from an extensive effusion of blood within the cranium, we may readily conceive that such a symptom would necessarily be developed. The moment such an effusion occurs, the brain is suddenly pressed down upon the arteries on which it rests, and also against every point of its bony case. It cannot, then, for want of room, rise and fall with the pulsations of the arteries at its base, as it does in its natural condition: and, this being the case, the mass of blood thrown from the heart at each contraction of its left ventricle would strike with great force against the compressed parts of the arteries, and communicate a shock to the brain which would be transmitted to, and be heard as an impulsive sound at, the surface of the cranium.

*American Journal of Med. Sciences. August,* 1838.

---

*On the Treatment of Delirium Tremens.* By JOHN WARE, M.D., Boston.

[THE following is an important practical document, which we recommend to the notice of our readers, as an appendix to the article on this disease in our last Number.]

The number of cases in private practice was 69, occurring during a period of about twenty years. Of these cases, 63 occurred among males and 6 among females. The whole number of deaths was 11; all the fatal cases were of males. Of 31 cases at the Almshouse, 5 were fatal. The ratio of mortality in all the cases was thus very nearly the same.

1. Eight cases were treated by *large doses* of opium, given with the intention of bringing about a termination of the paroxysm by sleep. The quantity administered varied, in different cases, from twenty-four to seventy-two grains, and it was usually given in the course of forty-eight hours. Four of these cases proved fatal. One died after sleep had been procured, the patient never awaking after the full effect of the remedy had been produced, but expiring in a state of coma. The remaining three died without having slept. Neither of these eight patients were bled. One of them wasthe subject of a severe acute disease, dysentery, in the course of which delirium tremens supervened: this was a fatal case. The others, so far as could be ascertained, laboured only under such general symptoms of disorder as are common to those made sick by intemperance, or some such chronic ailment as is frequent among persons of those habits, and could not be supposed to influence the course or event of the delirium. In the cases which recovered, restoration to health took place speedily and completely after sleep had taken place.

2. Seven cases were treated by *small doses* of opium, or opium given in such manner and quantity as not to have a distinct and powerful influence in the procuring of sleep; the quantity not exceeding two or three grains in twenty-four hours. Two of these patients died, both without having slept. One was labouring under severe peripneumony when attacked by delirium tremens: this case was fatal. One patient was bled, and this was one of the favorable ones.

3. Twelve cases were treated principally by repeated and continued vomiting, according to the mode of practice recommended by Dr. Klapp, of Philadelphia. Tartarized antimony was chiefly relied on for this purpose; but in a few cases the sulphate of copper and ipecacuanha were substituted, with no apparent difference in the effects of the treatment. Two of these patients laboured under severe disease, one of the brain and one of the cellular membrane around the knee-joint; the former died, the latter recovered. One patient was bled, and this recovered. Of the whole number, one died.

4. In two patients, a single copious bleeding from the arm was the only remedy employed, and in both the disease speedily gave way.

5. In nine cases, the mode of practice was what may be termed, for convenience of distinction, eclectic. The treatment was adapted to the prominent symptoms in each patient, having regard, in its application, rather to the general character of the case and the indications of derangement in particular organs, than to the presence of the peculiar affection of the brain which constitutes delirium tremens. Of course, a large proportion (seven) of these cases were decided cases of acute local disease, and were treated by the usual remedies. Five of the nine were bled; and of these, two died. Of the whole nine, three died; all of them being cases of peripneumony.

6. One case, in which the delirium accompanied erysipelas of the face and head, was treated by large doses of the sulphate of quinine. This recovered.

7. One case was treated by mercurials: salivation occurred, and the patient recovered.

8. In 29 cases, the mode of treatment was what may be properly denominated expectant. It is not intended to imply, however, that no remedies were administered. At the commencement of many of them, active measures were employed for a short period. Thus, some were bled, some leeched, to some an emetic was given, several were blistered upon the neck, and all were more or less subjected to the operation of cathartics. Besides these remedies at the outset, various articles were administered in the course of the several cases, but usually of an inefficacious character, or in such doses as probably to have had no influence on the course of the disease. For example, small doses of sp. ether. nit., liq. ammon. acet., tinct. hyoscyam., ext. conii, tinct. humuli, tinct. valerian, tinct. assafœtid., and various other medicines, were administered; but, from the amount and efficacy of the substances thus taken, no physician acquainted with their power would for a moment suppose them to have had any control over the disease.

All these cases were free from combination with acute disease, with one exception: in this there was inflammation of the arachnoid membrane of the brain, as determined by dissection. This was fatal. Four patients were bled, and all of them recovered. Of the whole number, 29, one died.

The results of the different modes of treatment will be more readily compared if they are thrown together into a tabular form.

| Treatment. | No. Cases. | Bled. | Died. | Recovered. | Complicated with acute Disease. |
|---|---|---|---|---|---|
| Opium, large doses..... | 8 | 0 | 4 | 4 | 1 |
| small ......... ... | 7 | 1 | 2 | 5 | 1 |
| Emetics .. ............... | 12 | 1 | 1 | 11 | 2 |
| Bleeding ............... ... | 2 | 2 | 0 | 2 | 0 |
| Eclectic .................... | 9 | 5 | 3 | 6 | 7 |
| Quinine ..................... | 1 | 0 | 0 | 1 | 1 |
| Mercurials .......... ..... | 1 | 0 | 0 | 1 | 0 |
| Expectant ............. .. | 29 | 4 | 1 | 28 | 1 |
| | 69 | 13 | 11 | 58 | 13 |

It appears from this statement that, of 15 cases in which opium constituted the principal remedy, 6 died; whilst, of 54 in which opium was not used at all, or only incidentally and in small quantities, only 5 died. Still further, if we separate from these 54 the 9 cases in which the treatment was eclectic, and in which the mortality seems to have arisen from the combination of acute disease, we have a remainder of 45 cases, of which only 2 were fatal. Again, if we compare the mortality of those cases in which opium was pushed to the full extent advised by writers on this disease with those in which no active remedy was employed, we have a mortality of 1 in 2 against a mortality of only 1 in 29.

This difference in the results of treatment would seem altogether too great to be attributed to accident, and goes far to establish the truth of the opinion formerly expressed, that opium given in large doses is actually injurious to patients labouring under delirium tremens. But, even admitting it as possible that the great proportion of fatal cases occurring where opium was used was accidental, it certainly, I think, will not be contended that the favorable termination of the cases not treated by opium was also owing to accident: and it will certainly follow that opium, if not absolutely injurious to these patients, is at least useless; and that our success in this disease will be sufficiently satisfactory without it.

*Boston Medical and Surgical Journal,* 1838.

